# Secretory immunity with special reference to the oral cavity

**DOI:** 10.3402/jom.v5i0.20401

**Published:** 2013-03-11

**Authors:** Per Brandtzaeg

**Affiliations:** Laboratory for Immunohistochemistry and Immunopathology (LIIPAT), Centre for Immune Regulation (CIR), University of Oslo, Oslo, Norway; Department of Pathology, Oslo University Hospital, Rikshospitalet, Oslo, Norway

**Keywords:** IgA, IgG, mucosa-associated lymphoid tissue (MALT), gut-associated lymphoid tissue (GALT), nasopharynx-associated lymphoid tissue (NALT), salivary glands, crevicular fluid, polymeric Ig receptor (pIgR), secretory component (SC), mucosal vaccination

## Abstract

The two principal antibody classes present in saliva are secretory IgA (SIgA) and IgG; the former is produced as dimeric IgA by local plasma cells (PCs) in the stroma of salivary glands and is transported through secretory epithelia by the polymeric Ig receptor (pIgR), also named membrane secretory component (SC). Most IgG in saliva is derived from the blood circulation by passive leakage mainly via gingival crevicular epithelium, although some may be locally produced in the gingiva or salivary glands. Gut-associated lymphoid tissue (GALT) and nasopharynx-associated lymphoid tissue (NALT) do not contribute equally to the pool of memory/effector B cells differentiating to mucosal PCs throughout the body. Thus, enteric immunostimulation may not be the best way to activate the production of salivary IgA antibodies although the level of specific SIgA in saliva may still reflect an intestinal immune response after enteric immunization. It remains unknown whether the IgA response in submandibular/sublingual glands is better related to B-cell induction in GALT than the parotid response. Such disparity is suggested by the levels of IgA in submandibular secretions of AIDS patients, paralleling their highly upregulated intestinal IgA system, while the parotid IgA level is decreased. Parotid SIgA could more consistently be linked to immune induction in palatine tonsils/adenoids (human NALT) and cervical lymph nodes, as supported by the homing molecule profile observed after immune induction at these sites. Several other variables influence the levels of antibodies in salivary secretions. These include difficulties with reproducibility and standardization of immunoassays, the impact of flow rate, acute or chronic stress, protein loss during sample handling, and uncontrolled admixture of serum-derived IgG and monomeric IgA. Despite these problems, saliva is an easily accessible biological fluid with interesting scientific and clinical potentials.

Ancient people recognized the antimicrobial properties of external body fluids and used topical application of saliva, colostrum, or urine as a cure or prophylactic (1). Many innate defense factors with a varying range of antimicrobial activities, such as lysozyme and lactoferrin, occur in exocrine secretions and may contribute to the barrier function of mucous membranes, in addition to the physical shielding properties of epithelia and mucin. All of these components of innate immunity cooperate intimately with adaptive humoral immunity mediated by antibodies.

Besredka proposed the existence of an external antibody system in 1919 when he showed that rabbits, after oral immunization with killed Shigella, were protected against fatal dysentery irrespective of the serum antibody titer (2). Over the last 20 years before his death in 1940, Besredka devoted most of his time to the study of mucosal immunization. In 1922, Davies supported Besredka's idea of a separate mucosal immune system when he detected antibodies against the dysentery bacillus in stools from infected patients several days before such antibodies appeared in serum (3). These and other pioneering studies on secretory immunity have been discussed by Besredka (4) and Pierce (5).

A molecular basis for secretory antibodies emerged in the 1960s when it was shown that saliva contains immunoglobulin (Ig) molecules (6). Conclusive evidence was not obtained, however, until the identification of different Ig classes was possible, and several laboratories reported that IgA predominates in most external secretions (7). The discovery in Tomasi's laboratory in 1965, showing that secretory IgA (SIgA) exhibits unique molecular properties, further intensified an investigation of mucosal immunity (8). SIgA was shown to be polymeric (mainly dimers) and covalently associated with an 80-kDa epithelial glycoprotein initially called ‘transport piece’ and later named ‘secretory component’ (SC). Furthermore, it was reported by Hereman's laboratory that the Ig class distribution of plasma cells (PCs) in the human gut differs strikingly from that in lymph nodes and bone marrow (9); in normal mucosal tissues, IgA^+^ PCs and their immediate precursors (plasmablasts) are approximately 20 times as numerous as IgG^+^ PCs.

In 1973, our laboratory provided the first direct evidence that human mucosal IgA^+^ PCs produce mainly dimers and perhaps some larger polymers (collectively called pIgA) rather than monomers (10), and in 1974 this characteristic was found to be associated with co-expression of a 15-kDa disulfide-linked polypeptide called joining (J) chain (11). In the late 1960s, we had observed that not only pIgA but also pentamers of IgM are preferentially transferred to external secretions such as saliva, apparently because of a common epithelial transport system (12, 13). Secretory IgM (SIgM) in parotid fluid was subsequently shown to be only non-covalently associated with SC (14), but in the gut epithelium IgM was found by immunoelectronmicroscopy to follow the same intracellular vesicular transfer route as pIgA and SC, while the secretory epithelial cells were devoid of IgG (15). A shared receptor-mediated mechanism involving endocytosis and transcytosis therefore seemed to exist for SIgA and SIgM formation (10, 11, 16, 17). Our transport model was based on a suggested crucial cooperation between J chain-expressing mucosal IgA^+^ and IgM^+^ PCs and SC-expressing serous-type of secretory epithelial cells (Fig. 1A).

**Fig. 1:**
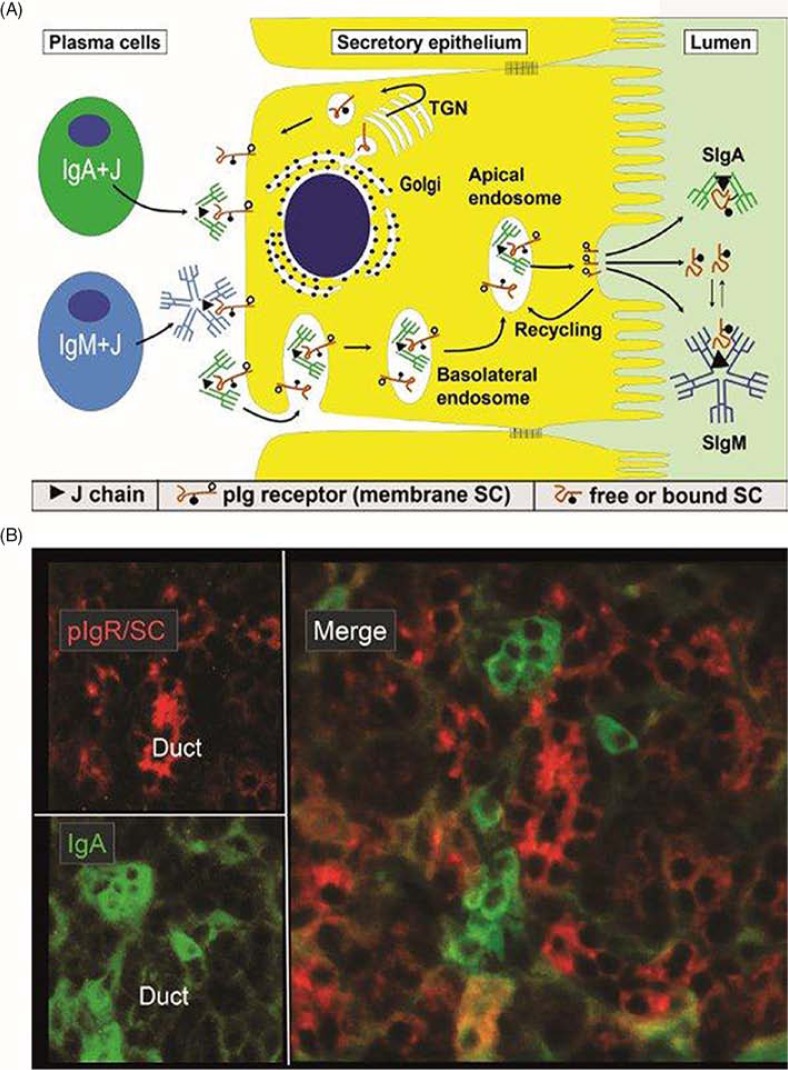
Receptor-mediated epithelial export of polymeric Igs (pIgs). (A) Model for local generation of secretory IgA (SIgA) and secretory IgM (SIgM). J chain-containing dimeric IgA (IgA+J) and pentameric IgM (IgM+J) are produced by local plasma cells (left). Polymeric Ig receptor (pIgR), or membrane secretory component (SC), is synthesized by secretory epithelial cell in the rough endoplasmic reticulum and matures in the Golgi complex by terminal glycosylation (•-). In the trans-Golgi network (TGN), pIgR is sorted for delivery to the basolateral plasma membrane. The receptor becomes phosphorylated (-o) on a serine residue in its cytoplasmic tail. After endocytosis, ligand-complexed and unoccupied pIgR is delivered to basolateral endosomes and sorted for transcytosis to apical endosomes. Some recycling from basolateral endosomes to the basolateral surface may occur for unoccupied pIgR (not shown). Receptor recycling also takes place at the apical cell surface as indicated, although most pIgR is cleaved to allow extrusion of SIgA, SIgM and free SC to the lumen. During epithelial translocation, covalent stabilization of SIgA regularly occurs (disulfide bond between bound SC and one IgA subunit indicated), whereas free SC in secretions stabilizes the non-covalently bound SC in SIgM (dynamic equilibrium indicated). Modified from Brandtzaeg (17). (B) Paired immunofluorescence staining for pIgR/SC (red) and IgA (green) in normal parotid tissue. The specimen had been washed in phosphate-buffered saline to extract diffusible extracellular proteins before fixation in cold ethanol (16). IgA-producing plasma cells are scattered among acini but most of them occur adjacent to intercalated ducts. Note that there is variable uptake of IgA both in acini and ducts as evidenced by peripheral and some cytoplasmic staining of the epithelial cells (original magnification: ×250).

## Biology of secretory immunity

Membrane SC is a carbohydrate-rich glycoprotein of ∼100 kDa constitutively expressed basolaterally on secretory epithelial cells (Fig. 1A), where it exhibits strong non-covalent affinity for J chain-containing pIgA and pentameric IgM (18). It belongs to the Ig supergene family with five extracellular domains and is now usually referred to as the polymeric Ig receptor (pIgR). Its human gene has been cloned and characterized (19), and several DNA elements could explain its remarkably high constitutive and cytokine-enhanced expression (20). Interferon-γ (IFN-γ) was the first cytokine shown to increase epithelial pIgR/SC expression and it was taken to be particularly responsible for the enhanced pIg export seen in concert with intensified local immune responses (21). IFN-γ-responsive DNA element in the upstream promoter and exon 1 of the pIgR gene have been identified (22), but there are also elements responsive to regulatory factors in the first intron (20). Altogether, both steroid hormones and proinflammatory cytokines can upregulate pIgR, including interleukin (IL)-17 which is particularly abundant at mucosal sites (23). Microbial components interacting with epithelial pattern recognition receptors (PRRs), such as toll-like receptors (TLRs), can do the same (24).

When pIgR reaches the apical surface of the epithelial cell, SIgA and SIgM are exocytosed after cleavage of the receptor; only its C-terminal segment remains for intracellular degradation (Fig. 1A). The extracellular part of pIgR (∼80 kDa) is exceptionally carbohydrate-rich (25), and when incorporated into the SIg molecules as bound SC it endows particularly SIgA (where it becomes disulfide-linked) with resistance against proteolytic degradation (26). Excess of unoccupied pIgR is released in the same manner by proteolytic cleavage to form so-called free SC according to the internationally recommended nomenclature (27). This 80-kDa glycoprotein can be found in most exocrine fluids including saliva (13), and on average approximately 50% of the exported SC occurs in a free form in various secretions (28). This ‘sacrificial’ nature of pIgR explains the need for its high level of constitutive expression (20). Importantly, both free SC and bound SC show several innate immune functions such as inhibition of epithelial adhesion of certain Gram-negative bacteria and neutralization of bacterial toxins (26). By equilibrium with bound SC (Fig. 1A), free SC also exerts a stabilizing effect on the quaternary structure of SIgM in which SC is only non-covalently linked (14).

The binding sites of pIgA and pentameric IgM initially contacting the first extracellular domain of pIgR have largely been defined (29). In addition, it has been shown that the J chain is crucial for the initial non-covalent complexing and stabilization between the Ig polymers and pIgR or free SC in *in vitro* experiments (18, 30). Thus, our original proposal that the J chain and pIgR/SC are involved in a ‘lock and key’ mechanism in the selective epithelial export of pIgA and pentameric IgM, is now firmly established (31–33). The J chain is normally produced preferentially by mucosal PCs (34), perhaps reflecting a recent generation of their precursors in germinal centers of mucosa-associated lymphoid tissue (MALT), while little or no J-chain expression would signify several precursor rounds through germinal centers according to the ‘decreasing potential’ hypothesis (35). However, the J chain can only become disulfide-linked to the Fc regions of IgA and IgM that carry a small tailpiece in their heavy chains (36). When it is produced by other PC classes (Table 1), it therefore remains in a free form and is degraded intracellularly without being released from the cells in detectable amounts (37, 38).

**Table 1:** J-chain positivity (%) of mucosal plasmablasts and plasma cells

	Ig class expression			
	
Exocrine tissue site	IgA	IgM	IgG	IgD
Mammary glands	94	100	56	100
Salivary and lacrimal glands	92	100	44 (72)*	95
Normal nasal mucosa	98	100	69	100
Normal small intestinal mucosa	99	100	87	ND

Based on published data from the author's laboratory.

* Data from IgA-deficient individuals.

## The involvement of salivary glands in secretory immunity

### Various origins of Igs in saliva

**Fig. 2:**
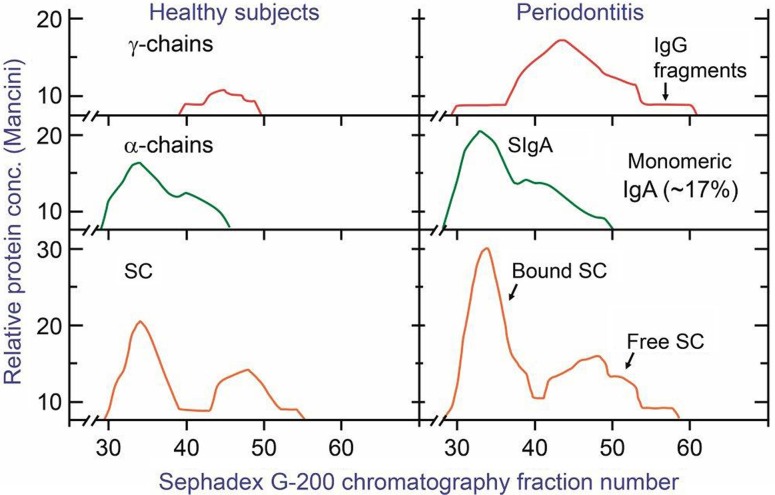
Elution patterns of Ig components in whole saliva after chromatography on Sephadex G-200 (column size, 2.5×37 cm; flow rate, 2.2 mL cm^−2^ h^−1^; fractions, 2.4 mL). Samples: 0.5 mL of 15 times concentrated unstimulated whole saliva from healthy individuals (left) or from patients with periodontitis (right). The distribution of Ig components was determined with specific antisera in single radial immunodiffusion (Mancini method) after three times concentration of the fractions. The squared diameters of the precipitin rings were used as concentration estimates. Modified from Brandtzaeg et al. (13).

The enzyme amylase is dominating in saliva (39, 40) so IgA does in fact represent a minor fraction of total salivary protein (13). However, the parotid IgA-to-IgG concentration ratio is about 500 times increased compared with that in serum (Table 2) as a result of selective epithelial pIgA export (Fig. 1A, B). The same transport mechanism also explains that the IgM-to-IgG ratio is substantially increased in normal parotid fluid compared with that in serum; but because of the diffusion advantage through epithelial basement membranes of the relatively small IgG molecule (41), pIgR-mediated salivary secretion of IgM is largely masked (12, 13). Much of the IgM in whole saliva seems to be explained by crevicular leakage as its level (in contrast that of IgA) is significantly related both to the serum IgM concentration and periodontal inflammation (13, 40). The monomeric fraction of salivary IgA is generally small – that is, about 10% in parotid fluid and 13–17% in whole saliva, depending on the clinical state of the gingiva (Fig. 2). It has been estimated that up to 77% of monomeric IgA in saliva is derived from serum and not from glandular PCs (42), although some of these cells produce a mixture of polymers and monomers, as discussed below (43).

**Table 2:** Variations in mean results of salivary IgA determinations performed by the same laboratory (LIIPAT, 1970–91)

Samples (No. of adult subjects)	Conc. (µg/mL)		IgA secretion rate (µg/min)

	IgA	IgG	
Stim. parotid secretion (n=9)^a^	40	0.36	27
Stim. parotid secretion (n=27)^a^	36	ND^c^	34
Stim. parotid secretion (n=19)^b^	27	ND	14
‘Unstim.’ parotid secretion (n=5)^a^	120	ND	10
‘Unstim.’ whole saliva:			
Healthy individuals (n=8)^a^	194	14.4	ND
Periodontitis patients (n=13)^a^	371	69.7	ND

Based on published data from the author's laboratory.

a Single radial immunodiffusion

b ELISA

c ND=not determined.

**Fig. 3:**
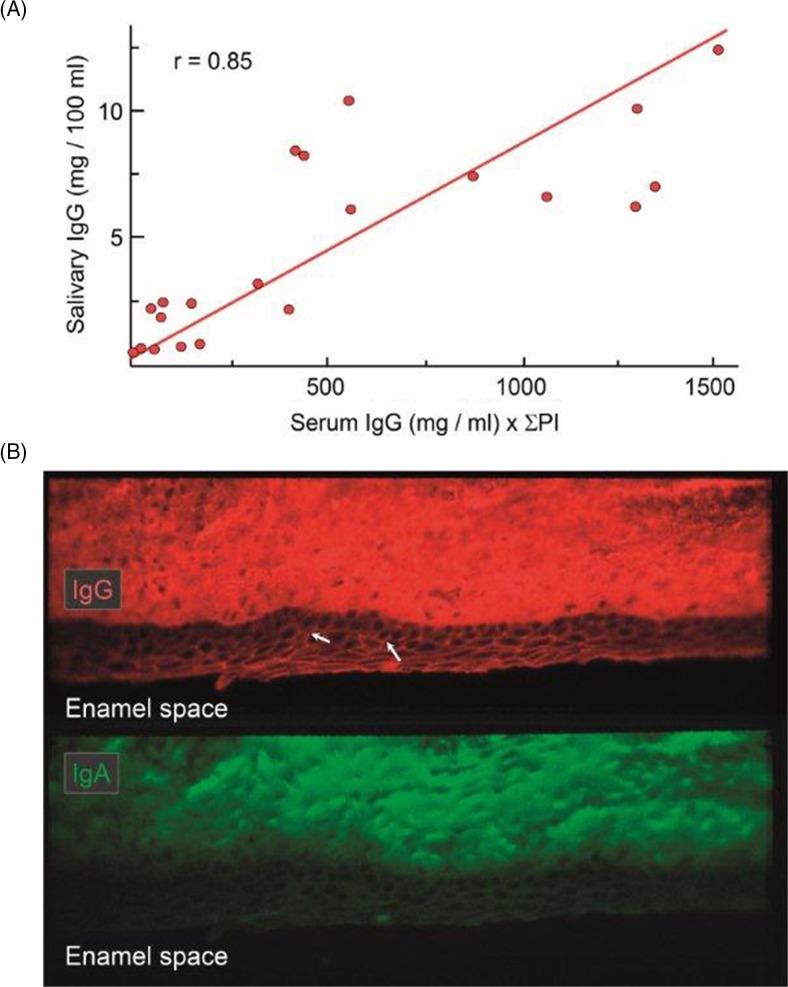
Distribution of serum IgG in whole saliva and crevicular epithelium. (A) Regression line for the relationship between concentrations of IgG in whole saliva and the corresponding serum concentrations multiplied by the sum of the individual's periodontal index (PI) scores. Adapted from Brandtzaeg et al. (13). (B) Immunofluorescence demonstration of IgG (red) and IgA (green) in monkey gingiva that had been directly alcohol-fixed *in situ* before being removed from the tooth. Middle part of the crevicular epithelium is shown. IgG and IgA permeate the connective tissue and particularly IgG appears intercellularly in the epithelium (arrows) (original magnification: ×125). Unpublished experiments (Brandtzaeg P and Tolo K).

These observations, and the significant association of the IgG concentration in whole saliva with the product of the serum level of IgG and the extent of gingival/periodontal inflammation (Fig. 3A), shows that IgG (and therefore also monomeric IgA of similar molecular size) mainly enters the oral cavity from the peripheral blood circulation via crevicular fluid (13, 40). Paracellular leakage of IgG (and IgA) through the crevicular epithelium can be observed *in situ* (Fig. 3B) and, by taking serum albumin as a reference, it has been estimated that <17% of IgG and <8% of IgA in crevicular fluid collected from periodontitis lesions is produced by local PCs in the gingival lesion (44). Thus, at least 95% of the IgA normally appearing in saliva is produced by PCs in the various salivary glands and transported into salivary fluids as SIgA dimers or larger polymers (Fig. 1A, B).

### Salivary IgA levels are highly variable

Quantitation of salivary IgA is afflicted with many methodological problems and is difficult to standardize (40). Moreover, there are significant variables in the collection, processing and storage of samples. Such problems are reflected in different values obtained for salivary IgA concentrations, even in studies performed by the same laboratory (Table 2). Thus, the results vary strikingly between measurements based on single radial immunodiffusion, ELISA or other methods, such as particle-enhanced nephelometric immunoassay (45). Because of the slow development of the salivary IgA system in our part of the world compared with underprivileged countries (46–48), age and geography are also important variables. Moreover, various stressors reportedly (49) influence the IgA levels in different manners (Table 3).

**Fig. 4:**
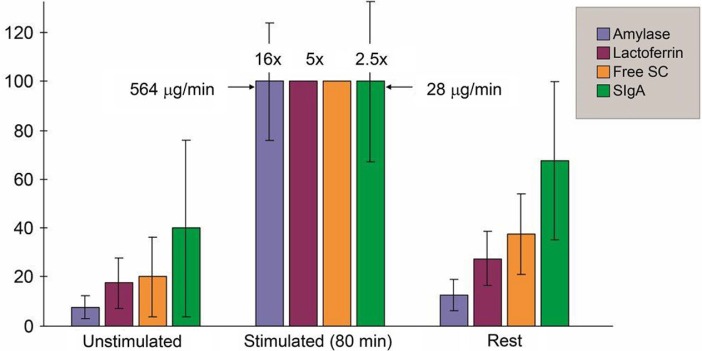
Secretion rates (µg/min/gland) of amylase, lactoferrin, free secretory component (SC), and secretory IgA (SIgA) in parotid fluid obtained before (unstimulated), during (stimulated), and after (rest) 80 min of acid gustatory stimulation of the secretion. Maximum average secretion rate in five subjects is for each component given as 100% on the vertical axis. Free SC and lactoferrin were undetectable in some stimulated samples, and data for these components are based on only four or three samples, respectively. Absolute figures (µg/min) and standard deviations (vertical lines) are therefore not indicated for the corresponding group maxima, while the average response for each component is indicated above the columns. Adapted from Brandtzaeg (39).

It is important to be aware of the striking impact of the secretion flow rate on the salivary IgA level (Table 2), which partly explains differences among studies. ‘Unstimulated’ parotid secretion thus contains at least three times more IgA than the stimulated counterpart (39, 50, 51), and a similar proportional difference has been reported for whole saliva (52). Some investigators have tried to avoid this problem by reporting salivary IgA related to total protein (dominated by amylase) or albumin; but this will also be misleading because the secretory response of individual salivary proteins is quite different (Fig. 4), with large individual variations (39, 53), and the salivary level of albumin will depend on leakage from serum in a manner similar to IgG and monomeric IgA, as discussed previously.

In some studies, the output of salivary proteins has been reported in secretion rates (µg/min), whereas in others the actual protein concentration (mg.L^−1^ or µg/mL) has been considered a better alternative for salivary IgA (54). Stimulated secretion may be preferable as a test sample (51, 54); it is more easily collected and less adversely affected by storage than unstimulated fluid (39), but these apparent advantages may not be valid when using collection swabs like that provided by Salimetrics^®^. We have recently obtained preliminary results for specific salivary IgA antibody levels after enteric immunization of volunteers with enterotoxigenic *Escherichia coli* (ETEC), implying that total salivary IgA may be concurrently elevated because of induction of unknown specificities (‘natural’ IgA antibodies); the intestinal immune response reflected in saliva as specific IgA could hence be partly masked by ‘normalizing’ the specific titer to total IgA in the same sample (54). It is not surprising that the introduction of a new bacterium in the gut causes ‘bystandard’ activation of B cells of unknown specificities which might migrate to the salivary glands. Thus, experiments in mice have shown that intestinal IgA resulting from exposure to one new commensal bacterium contained less that 10% detectable specific antibodies. Experiments in our laboratory with chronic stimulation by a single protein antigen resulted in induction of at least 60% PCs of unknown reactivity co-localized with the specific ones (44).

Although the secretion rate of parotid IgA appears to be more stable over time than the actual IgA concentration (13, 39), SIgA is more subjected to short-term variation than other salivary proteins (55, 56). This may reflect differences in the glandular structures involved in the secretion of the various protein components (57) and also the fact that SIgA is mainly a product of adaptive immunity (Fig. 4). Thus, studies in inbred mice of the same age suggested that fluctuations in glandular IgA^+^ PCs contributed to a striking individual variation in total salivary IgA levels over time (58), in accordance with our observations after enteric vaccination in humans discussed above (59). Diurnal and seasonal variations should also be considered, as should relation to meals, cigarette smoking, and hormonal effects such as differences between men and women (45) and those caused by pregnancy (60), in addition to various stressors as mentioned above (Table 3).

**Table 3:** Effect of different stressors on salivary IgA levels

Definition of stressors	Salivary IgA
Chronic academic stress (e.g. during exam period)	Reduced
Acute academic stress (e.g. just before or after exam)	Increased
Acute ‘naturalistic stress’ (e.g. work shift)	Increased
Laboratory stressors:	
‘Acute coping’ of challenges (sympathetic activation followed by parasympathetic rebound)	Reduced (?) Increased
‘Passive coping’, feeling of disgust	Reduced

Adapted from Bosch (49).

Standardized Ig quantification is even more challenging in whole saliva than in parotid or sublingual/submandibular fluids (45). First, the contributions to whole saliva from the minor and various major glands vary greatly according to the flow rate (61), and contamination with nasal secretions (and tears) may be difficult to avoid, particularly in uneasy children (62). Notably, the mean IgA concentration in secretion from labial glands has been reported to be three times higher than that in parotid fluid (63), and the buccal glands seem to be even more active in 3-year old children (64). On this basis it has been estimated that the minor glands contribute 30–35% of the total salivary IgA (63), but problems in collecting these secretions limit this approach, such as evaporation of small samples. Second, the flow rate of whole saliva cannot be accurately measured. Third, whole-saliva samples usually require centrifugation before quantification, and the sediment represents a variable IgA loss. Thus, even in parotid secretion a substantial proportion (50–60%) of IgA exists in >25S complexes, and this fraction is even higher for SIgA dissolved from the sedimented mucin clot obtained by centrifugation (65), most likely reflecting the mucophilic properties of bound SC (26). In whole saliva there is the additional problem of IgA binding to oral bacteria (66) but centrifugation may be avoided by cautious suction of the fluid from the floor of the mouth or by controlled sampling with swabs or absorbing discs (67).

Despite the various disadvantages, whole saliva is commonly used as a ‘representative’ external secretion because it is easily obtained. To increase fluid volume, chewing on paraffin wax or Parafilm is often applied. Although this is convenient, it entails certain pitfalls; the wax adsorbs organic material (68) and the chewing may increase leakage of serum proteins into the oral cavity, particularly from inflamed gingivae. Therefore, oral health must always be carefully considered when whole saliva is used for immunological investigation, but this has rarely been the case. When we studied a pIgR/SC-deficient mouse strain generated in our laboratory, we noted that touching the oral or the intestinal mucosa with a wick for SIgA sampling was sufficient to cause epithelial bulk leakage of proteins from tissue fluid (69).

Altogether, although numerous studies of IgA antibodies in saliva from healthy individuals and after parenteral or oral (enteric) vaccination as well as after various infectious diseases have been published, it may be questioned how reliable or meaningful some of the results are. Many of the early reports are summarized in a previous review (44).

### Production and export of pIgs in salivary glands

**Fig. 5:**
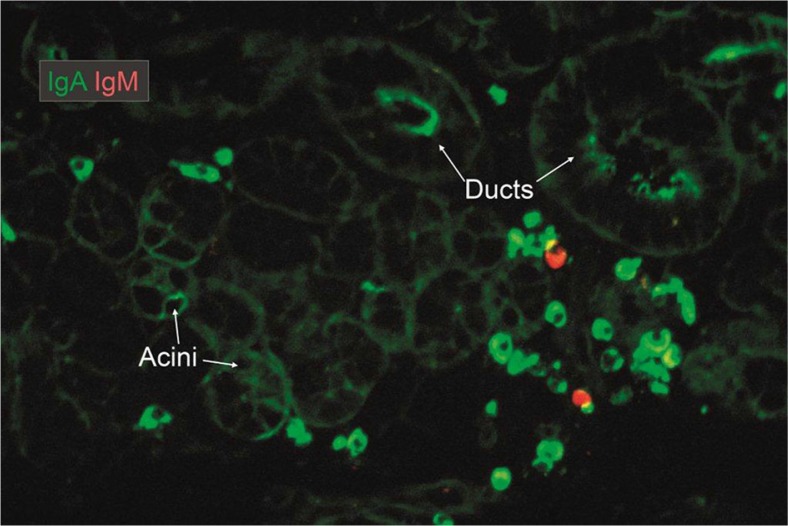
Merge of paired immunofluorescence staining for IgA (green) and IgM (red) in section of saline-extracted and ethanol-fixed specimen (16) of normal submandibular salivary gland. There is a dominance of IgA-producing plasma cells mainly adjacent to ducts. Note that there is a variable uptake mainly of IgA both in acini and ducts as evidenced by peripheral and some cytoplasmic staining of epithelial cells, but the luminal ring in striated duct at the middle top might represent adherent IgA from the secretion (original magnification: ×125).

**Fig. 6:**
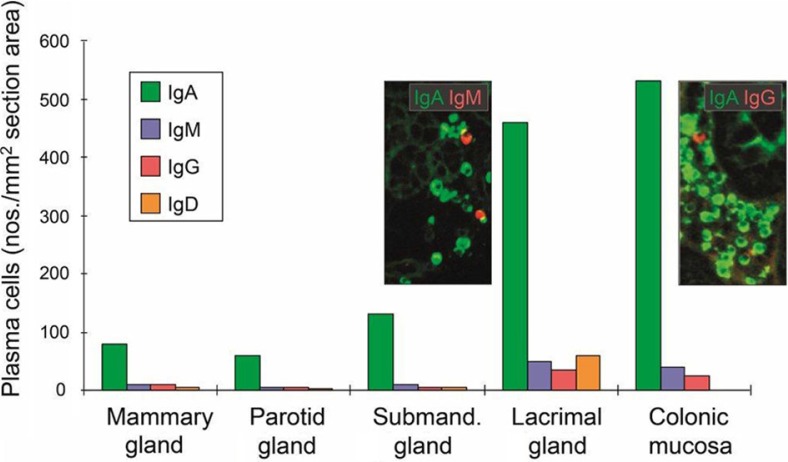
Average tissue densities of plasmablasts and plasma cells producing different Ig classes at various normal secretory sites as indicated. Representative areas illustrating merge of immunofluorescence staining (see color key) are shown for submandibular gland and colonic lamina propria. Based on published data from the author's laboratory.

Local IgA^+^ PCs occurs scattered among acini of histologically normal major salivary glands and are often seen in clusters adjacent to ducts (Fig. 1B). Notably, the submandibular glands (Fig. 5) contain on average approximately twice as many IgA^+^ PCs per tissue unit as the parotid (70, 71), in accordance with a larger export of SIgA (54). It is tempting to speculate that antigens gain easier access to sublingual/submandibular glands, thereby inducing a more active local immune system. Interestingly, the daily output of IgA/kilogram wet weight of lactating mammary glands (minus fatty tissue) is similar to the average output for salivary glands, so the superiority of the former as an SIgA source depends on the organ size and ductal storage system (71). Thus, the average stromal density of IgA^+^ PCs is similar in the parotid and lactating mammary glands (72), but it is six to seven times less than in lacrimal glands and intestinal mucosa (Fig. 6).

**Fig. 7:**
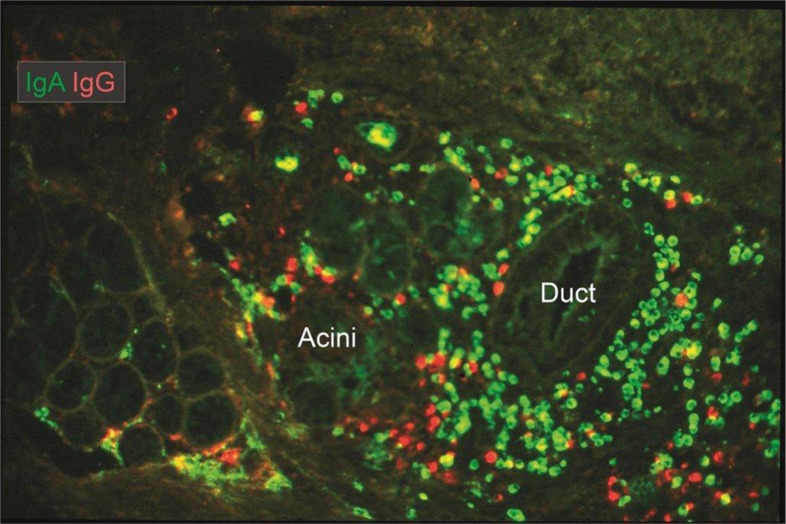
Merge of paired immunofluorescence staining for IgA (green) and IgG (red) in section of saline-extracted and ethanol-fixed specimen (16) of buccal minor salivary gland. Note that there is a relatively dense population of plasma cells compared with major salivary glands (cf. Fig. 5), and that IgG-producing cells are quite abundant at the periphery. Both ducts and serous parts of acini show faint selective staining for IgA, reflecting external transport (original magnification: ×80).

Because the minor salivary glands are numerous and have close proximity to a mucosal surface (like the intestinal and lacrimal glands), they may be relatively abundantly exposed to exogenous antigens and are probably important in the defense of the oropharynx. This possibility is supported by the observation of numerous IgA^+^ PCs adjacent to their ducts (73) and the comparatively high output of SIgA from these glands (63). In fact, the density of IgA^+^ PCs in minor salivary glands (Fig. 7) has been reported to be three times that in the parotid (74). Because salivary gland ducts express MHC class II (HLA-DR) molecules, the possibility exists that the ductal epithelium may be involved in antigen presentation and then contribute to local terminal differentiation to IgA^+^ PCs (75).

**Fig. 8:**
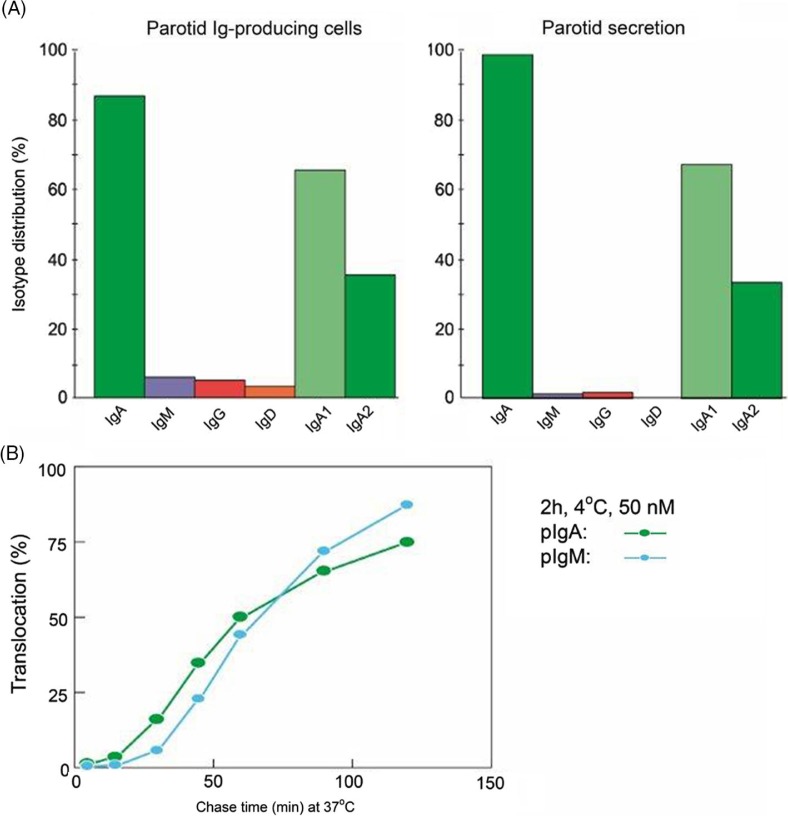
Relationship between local production of Ig isotypes by parotid plasma cells and Ig transfer by secretory epithelium. (A) Compared with the local production, export of IgA into stimulated parotid secretion is clearly favored over export of IgM (and IgG and IgD), whereas translocation of the two subclasses of IgA appears to be handled equally well by the glandular epithelium. (B) Comparison of epithelial translocation of dimeric IgA (pIgA) and pentameric IgM (pIgM) was performed *in vitro* with polarized MDCK cells transfected with the human polymeric Ig receptor. Cells were incubated with ^125^I-labelled pIgA or pIgM for 2 h at 4^o^C, washed for 10 min at 4^o^C, and chased at 37^o^C for different times as indicated. Translocation is expressed as the cumulative appearance of ^125^I-pIgA and ^125^I-pIgM in the apical medium. Each point represents mean result of three filters for pIgA and pIgM translocation at 50 nM ligand concentration. Adapted from Norderhaug et al. (29).

As discussed previously, SIgM is not secondarily stabilized by bound SC through disulfide bonding (14), and its resistance to proteolytic degradation is inferior compared to SIgA. Also, when comparing the proportions of parotid PC classes and the IgA-to-IgM concentration ratio in the secretion (Fig. 8A), the glandular export of pIgA is favored over that of pentameric IgM by a factor of approximately five (or 12-fold on a molar basis) (41). This is not explained by different handling of the two polymers by pIgR (Fig. 8B) but is due to diffusion restriction for the relatively large IgM pentamer through stromal matrix and basement membranes, inhibiting its access to the basolaterally expressed pIgR (41). In fact, human pentameric IgM shows much higher affinity for free SC *in vitro* than does pIgA (30).

The subclass IgA2 is more stable than IgA1 because of its resistance to certain bacterial proteases (76). Therefore, it is interesting that a relatively large proportion (35–38%) of the IgA^+^ PCs in salivary glands produce IgA2 (77). In this respect, the salivary glands are intermediate between the upper airways and the distal gut, a disparity that clearly reflects regional immunoregulatory differences (34). In agreement with the similar affinity of IgA1 and IgA2 for free SC *in vitro* (30), both subclasses appear to be equally well exported by pIgR into the parotid secretion (65) (Fig. 8A).

**Fig. 9:**
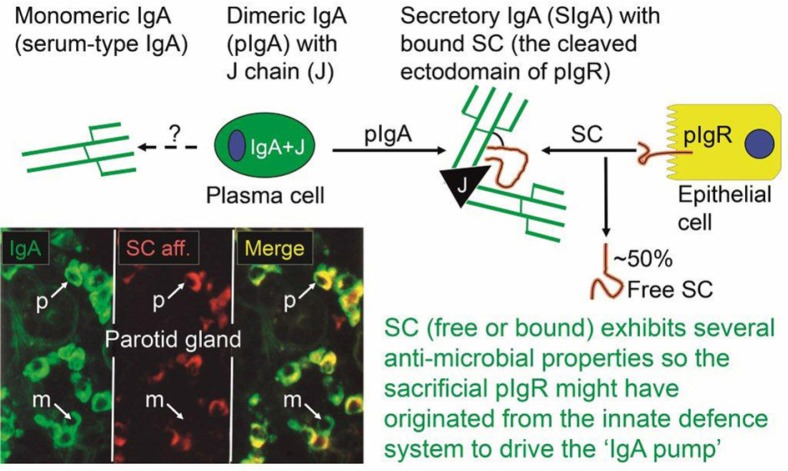
Generation of secretory IgA (SIgA) and free secretory component (SC). SIgA is formed as a hybrid antibody molecule stabilized by a disulfide bridge between the two cell products. The amount of dimeric IgA (pIgA) produced by a plasma cell depends on its level of J-chain expression, which generally is high in mucosal and glandular tissue. Inset (left) shows direct demonstration of abundant cytoplasmic expression of pIgA (p) in most parotid plasmablasts and plasma cells achieved by *in vitro* affinity test with free SC on tissue section as described (10), whereas a single cell producing only monomers (m) is seen in this field. On average, approximately 50% of SC occurring in various secretions is in a free form (unoccupied by ligand). The immunostained panel is from Brandtzaeg (43).

Evaluation of salivary-gland IgA^+^ PCs for J-chain expression and *in vitro* cytoplasmic affinity for free SC (marker of pIgA production), has indicated that almost 90% of them are variably involved in production of polymers (43). These are immediately available for the unique pIgR-driven epithelial transport system which generates both free SC and the hybrid SIgA molecule (Fig. 9), where the bound SC in a changed conformational shape covers most of the J chain, according to the most recent modeling studies (78). Thus, the many cartoons in the literature depicting bound SC wrapped around the Fc portions of the two IgA subunits are definitely wrong.

There have been many conflicting opinions about the expression of pIgR/SC in salivary glands. However, in our laboratory it has been localized by immunostaining mainly to the serous-type of epithelial cells (Fig. 1B); intercalated duct cells are usually more positive than acinar cells, while in striated ducts the expression is often limited to a luminal rim which may represent adherent SIgA (57, 72).

## Homing of activated B cells to salivary glands

### Multiple sites of mucosal B-cell activation

Initial immune stimulation to generate memory/effector B cells for mucosal pIgA responses takes place mainly in MALT structures, particularly Peyer's patches of the distal ileum and other parts of gut-associated lymphoid tissue (GALT), such as the numerous isolated lymphoid follicles and the appendix (34, 79). From these inductive sites, the activated B cells reach peripheral blood and migrate to secretory effector sites where they extravasate or are excluded on a competitive basis depending on complementary adhesion molecules and chemokine–chemokine receptor pairs (79, 80). The homing is successfully accomplished when it is directed by interactions between several dynamically regulated endothelial adhesion molecules or ‘addressins’ and the corresponding ligands (‘homing receptors’) expressed on the memory/effector cells. By such complex mechanisms, mucous membranes are furnished with locally produced secretory antibodies partly in an integrated way, ensuring a variety of specificities at every secretory site, but also in a compartmentalized manner making the secretory immune system less ‘common’ than previously believed (27, 34, 79, 81).

It is not well delineated which MALT structures are most important for induction of immune responses subsequently expressed as salivary IgA antibody production, but there is convincing evidence both in animals and humans that activated B cells migrate from GALT to salivary glands (82–85). Thus, the production of IgA antibodies to gluten (gliadin) that is induced in the gut of patients with coeliac disease, is reflected as diagnostic IgA titers in whole saliva (86, 87). Nevertheless, in subjects immunized orally with a cholera toxin (CT) B subunit–whole-cell *Vibrio cholerae* vaccine, the specific IgA antibody detection sensitivity in whole saliva was not better than in serum and only approximately 50% of that in intestinal lavage (88). After infection with *V. cholerae* or enterotoxigenic *E. coli*, the detection sensitivity for antibodies against the respective toxins increased, but not so much as that seen in serum (88). These results suggested that enteric immune induction is not so well reflected in the salivary IgA system. This was not due to prior adjustment of the specific antibody levels to the total salivary IgA levels because such normalization was not performed. However, in this study the enteric–oral B-cell homing axis might have been partly masked because CT and enteropathogens breach the gut epithelium and are excessively disseminated to the systemic immune system, as reflected by the high serum levels of specific IgG antibodies (88).

Recent studies point to the possibility that nasopharynx-associated lymphoid tissue (NALT), such as the adenoids and palatine tonsils in humans (27), may be more important than GALT as an inductive site for memory/effector B cells destined to salivary glands (89, 90). Although it remains uncertain to what extent these lymphoepithelial structures of Waldeyer's ring are functionally comparable to rodent NALT (91), they are strategically located to orchestrate mucosal immunity against both airborne and alimentary antigens. Moreover, they are well designed for immune induction because of their deep and branched antigen-retaining crypts and the absence of antigen-degrading digestive enzymes. Rodent NALT consists of paired lymphoid cell aggregates present at the entrance of the nasopharyngeal duct and exhibits structural similarities to GALT with no crypts (92); its inductive function may be important for immunity of the upper aerodigestive tract, including the salivary glands (91). Organized duct-associated lymphoid tissue (DALT) has been described in minor salivary glands of monkeys, but there is no experimental evidence for its putative local immune-inductive capacity, and similar structures have not been observed along the ducts of human salivary glands (93).

**Fig. 10:**
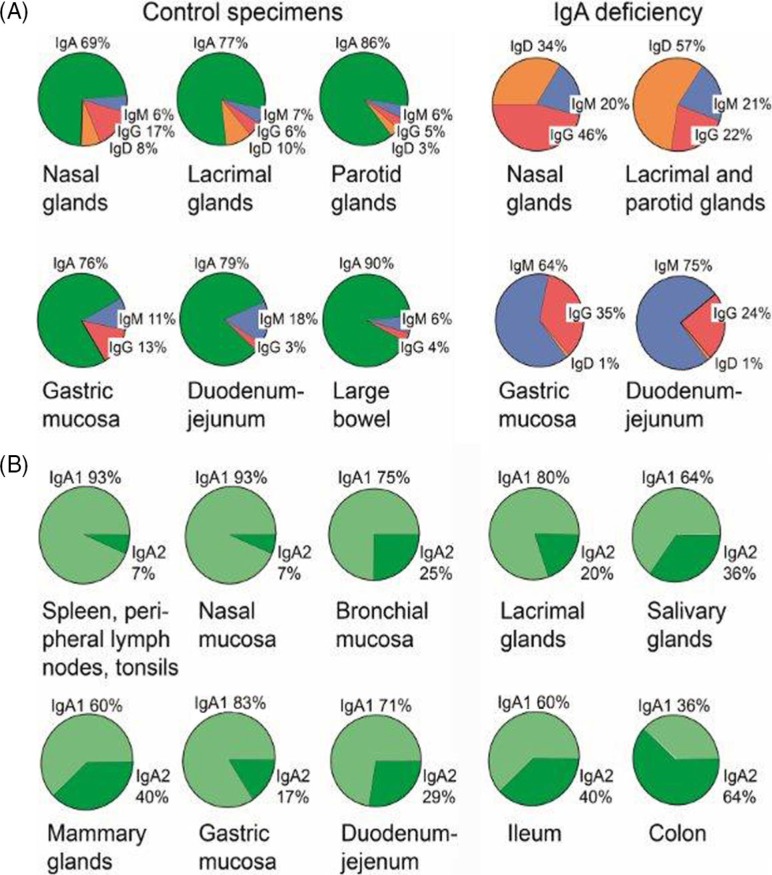
Relative production of Ig classes and IgA subclasses at human secretory effector sites. (A) Average percentage distribution of plasmablasts and plasma cells producing different Ig classes in various human secretory tissues from healthy controls and subjects with selective IgA deficiency. Based on published data of the author's laboratory. (B) Average percentage subclass distribution of IgA-producing plasmablasts and plasma cells in human lymphoid and normal secretory tissues. Based on published data from the author's laboratory except data for bronchial mucosa. Adapted from Brandtzaeg and Johansen (34).

Overall, it remains to be conclusively shown whether regional antigen stimulation results in better salivary immune responses than immune induction in GALT, although this has been suggested by tonsillar immunization with streptococci in rabbits (94). Moreover, there is circumstantial evidence to imply that the immune system of the human gut differs considerably from that of the upper aerodigestive tract with regard to B-cell precursor sources and/or immunoregulatory events (34, 89). First, a striking disparity exists between the two regions in terms of local IgD responses, particularly in IgA deficiency (Fig. 10A). Second, there is also a disparity for the subclass distribution of IgA^+^ PCs, as mentioned previously (Fig. 10B).

### Evidence for homing of memory/effector B cells from NALT to salivary glands

**Fig. 11:**
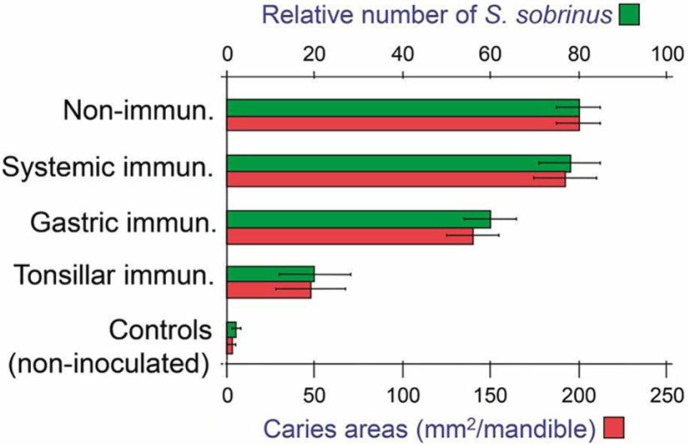
Effect of tonsillar immunization with killed *Streptococcus sobrinus* on experimental bacterial tooth colonization and dental caries in a rabbit model. Comparison is made with results obtained in non-immunized (top) or non-inoculated (bottom) rabbits and after systemic or intragastric (enteric) immunization. Adapted from Fukuizumi et al. (94).

As alluded to above, compartmentalization of the mucosal immune system is supported by preferential appearance of protective IgA antibodies in saliva of rabbits after tonsillar rather than enteric application of *Streptococcus sobrinus* in a dental caries model (Fig. 11). Likewise, in an *S. mutans*-based caries model in rats, intranasal immunization with a recombinant bacterial fusion protein induced salivary IgA antibodies and serum IgG and IgA antibodies (95). Also, direct immunization of human palatine tonsils, and particularly nasal vaccination, gave rise to local B-cell responses in palatine tonsils and adenoids as well as circulating specific B cells which apparently were excluded from the intestinal mucosa (96). Of further note, infants dying of sudden infant death syndrome (SIDS) were found to have overstimulated tonsillar germinal centers reflected by an increased number of IgG^+^ and IgA^+^ PCs (97), probably caused by airway infection; and such activated B cells were apparently distributed in excessive numbers to regional secretory effector sites, including the parotid glands (98), thereby giving rise to increased levels of salivary IgA and IgM in SIDS (99).

**Fig. 12:**
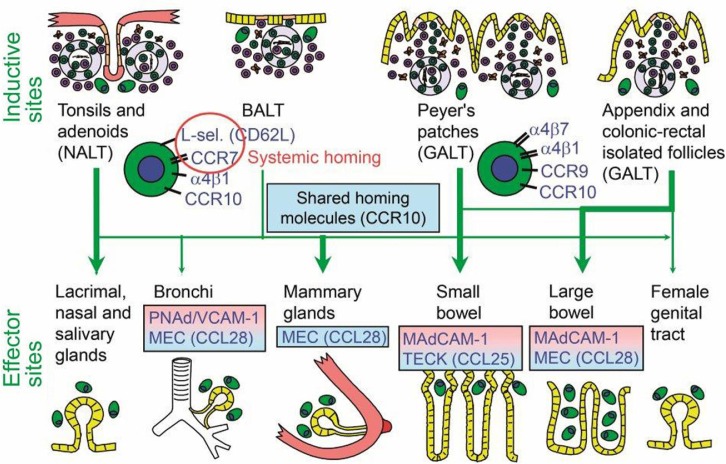
Homing properties of human mucosal memory/effector B cells. Putative scheme for compartmentalized migration of B cells from inductive (top) to effector (bottom) sites. Depicted are more or less preferred pathways (graded arrows) presumably followed by mucosal B cells activated in nasopharynx-associated lymphoid tissue (NALT) represented by palatine tonsils and adenoids, bronchus-associated lymphoid tissue (BALT), and gut-associated lymphoid tissue (GALT) represented by Peyer's patches, appendix, and colonic-rectal isolated lymphoid follicles. The principal homing receptor profiles of the respective B-cell populations, and adhesion/chemokine cues directing extravasation at different effector sites, are indicated (pink and blue panels) – those operating in lactating mammary glands apparently being shared for NALT- and GALT-derived cells. Homing molecules integrating airway immunity with systemic immunity are encircled in red. Adapted from Brandtzaeg (130).

Evidence is therefore accumulating to support the notion that NALT supplies secretory effector sites of the upper aerodigestive tract with activated pIgA^+^ precursor cells (89, 100). One reason for the suggested homing dichotomy between this region and the small intestine appears to be differences in the employed homing molecules (80). The leukocyte integrin α4β7 is important for B-cell extravasation into the gut lamina propria by interaction with the mucosal addressin cell adhesion molecule (MAdCAM)-1 expressed on the intestinal microvascular endothelium (Fig. 12); but this integrin does not appear to be important for homing to the airways and salivary glands (34). Also the involved chemokine receptor–chemokine interactions (CCR9–CCL25 *versus* CCR10–CCL28) show a striking dichotomy between the two body regions (81). The low expression level of gut-homing molecules after NALT immunization, particularly α4β7, has been shown in mice to be the direct reason for exclusion of the activated B cells from small intestinal mucosa (80). Human NALT induction instead induces α4β1 interacting with vascular cell adhesion molecule (VCAM)-1 and also the expression of the systemic homing molecules L-selectin (CD62L) and CCR7 (34, 81, 89). This probably reflects that palatine tonsils and adenoids act as a ‘cross-road’ between mucosal and systemic immunity (Fig. 12).

Sublingual immunization in mice with stimulation of B cells in cervical lymph nodes apparently results in dissemination of immunity by the same homing molecules as those operating after NALT stimulation, but may be a safer approach with no possibility for redirecting antigens/adjuvants to the brain (101). Sublingual instead of subcutaneous allergen administration is an established alternative approach to desensitize pollen-allergic patients, and such sublingual immunotherapy (SLIT) is now being tested also for food allergens. Interestingly, in a SLIT trial for peanut allergy, six out of 10 patients showed significant induction of IgA (and SIgA) peanut-reactive antibodies in saliva (102).

Attempts have been made to support the NALT concept in humans by evaluating the effect of adenotonsillectomy on the regional SIgA levels. The pioneering report by Ogra (103) in 1971 showed that combined tonsillectomy and adenoidectomy in children reduced the level of IgA antibody to poliovirus three- to four-fold in their nasopharyngeal secretions and delayed or abrogated their local immune response to subsequent live polio vaccine. Jeschke and Ströder (104) performed tonsillectomy in children and found that their serum Ig and salivary IgA decreased for up to 3 years. D'Amelio et al. (105), however, observed no salivary IgA reduction (but decreased serum IgA) in previously tonsillectomized adults (16–24 years old). Conversely, Cantani et al. (106) found that salivary IgA as well as serum IgA (and less so IgG and IgM) were significantly reduced 4 months after adenotonsillectomy in children. Subsequently, however, studies in tonsillectomized children showed instead elevated salivary Ig levels after 3–4 years (107) whereas no effect was found in tonsillectomized young adults after 6 months except for a slight reduction of total IgM and salivary IgG antibodies to *S. mutans* and Epstein Barr virus (108).

It has to be concluded that there is a need for more extensive clinical studies, perhaps focusing collectively on the adenoids/palatine tonsils and cervical lymph nodes as inductive lymphoid tissue for regional immune responses (89). This notion is supported by reports suggesting reduced salivary IgA levels in children with recurrent tonsillitis (109) or with adenoid hyperplasia (110). Decreased J-chain expression is a consequence of recurrent tonsillitis, and to a lesser extent adenoid hyperplasia, implying that chronic inflammation may compromise the potential of human NALT to furnish the regional SIgA system with pIgA^+^ plasmablasts (89).

## Development of the mucosal immune system

### Effect of age on salivary Ig levels

Ontogeny has a striking impact on salivary Ig levels, and age-matched control groups must therefore always be available when quantitative studies are performed. IgG is initially predominating in saliva of the neonate– being derived from interstitial tissue fluid in which maternal IgG abounds in the perinatal period as a result of placental transfer (47). The levels drop in parallel with albumin, so after 2–3 months very little IgG is present in whole saliva (111). A postnatal decrease of permeability thus takes place in the oral mucosa similar to the so-called ‘gut closure’; catabolism of the acquired maternal IgG is also involved.

A considerable proportion (approximately 50%) of infants have detectable IgD in their whole saliva during the first months of life (111), and IgD is also seen, though with decreasing frequency, over the next five months – that is, during the time period when relatively many IgD^+^ PCs occur in salivary glands (46). The locally produced IgD most likely reaches saliva by paracellular diffusion due to the neonatal epithelial permeability.

**Fig. 13:**
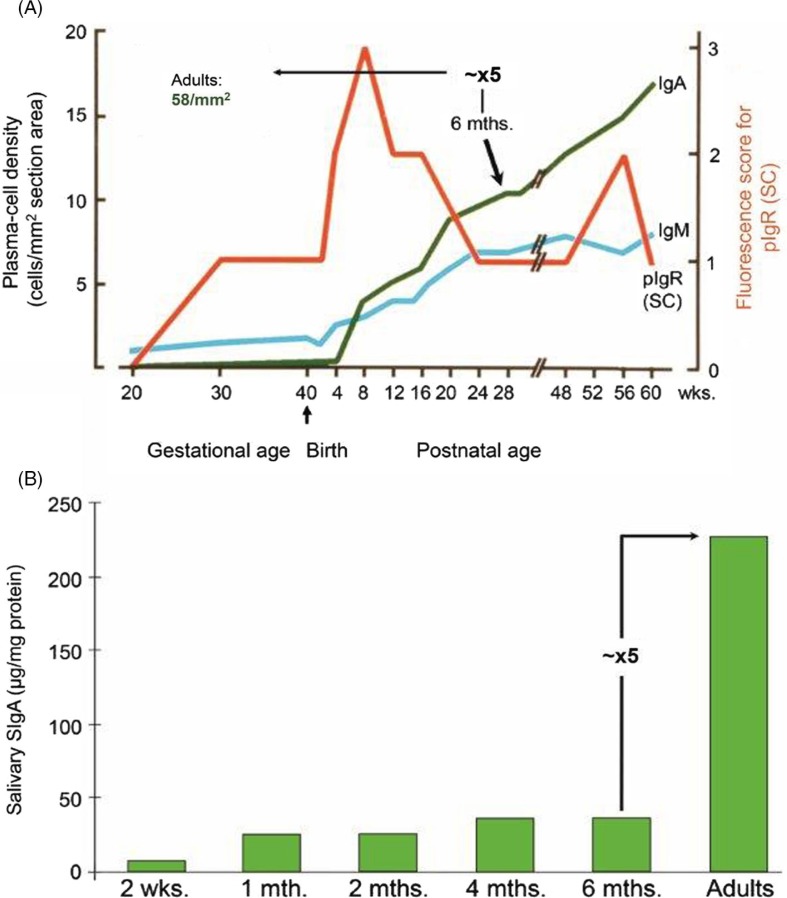
Development of the secretory IgA (SIgA) system in parotid glands and saliva. (A) Ontogeny of Ig-producing cells and epithelial expression of the polymeric Ig receptor (pIgR/SC) in parotid glands. Based on data from Thrane et al. (46). (B) Ontogeny of SIgA as determined in unstimulated whole saliva. Based on data from Fitzsimmons et al. (114). Note that both the density of IgA^+^ plasmablasts and plasma cells as well as the SIgA level at about 6 months of age has to increase approximately 5 times to match the adult values.

Low levels of salivary SIgA and SIgM antibodies to *E. coli*, and occasionally to poliovirus, have been reported to be present in Swedish infants during the first days of life (112), although it is difficult to detect SIgA and SIgM as such in saliva at this early age (47, 111). Good evidence exists for a fetal origin of these antibodies and the antigenic stimuli might be maternal anti-idiotypic IgG (112). Salivary SIgA and SIgM antibodies to cow's milk proteins have likewise been detected at birth, perhaps being elicited *in utero* by a similar mechanism or by the presence of dietary antigens in amniotic fluid to which the fetal oral cavity is continuously exposed (47). The cellular basis for some production of SIgA is present in fetal salivary glands (46). Occasional IgA^+^ and IgM^+^ PCs occur as early as from the 20th gestational week, the latter class dominating markedly; 1 month after birth their numbers start to increase rapidly, the IgA class becoming predominant at about 2 months and approaching the lower normal adult range at around 15 months (Fig. 13A) while the subsequent increase throughout early childhood seems to be small (47).

Most of the fetal salivary gland IgA^+^ PCs (∼90%) are of the IgA1 subclass and virtually all express J chain (46). However, during the first 3 months after birth the IgA1-to-IgA2 PC ratio in salivary glands approaches the normal adult value (Figs. 8A and 10B). This might reflect an increasing postnatal influx of IgA^+^ precursor cells from GALT where the IgA2 isotype normally predominates (77, 113). At the same time the high level of J-chain expression (94–97%) is maintained for both subclasses of PCs (46) attesting to their production of pIgA ready for export. Accordingly, a similar change in the IgA1-to-IgA2 concentration ratio has been observed in infant saliva (114).

Expression of pIgR/SC occurs in a few acini and small ducts of salivary glands at the 20th week of gestation, increasing markedly up to the 30th week (46). As reviewed elsewhere, a similar expression pattern has also been observed by others (47). Apical staining for IgA in epithelial elements has been noted after the 30th week of gestation, suggesting external transport of SIgA. Interestingly, there is a temporarily elevated epithelial expression of pIgR/SC shortly after birth (Fig. 13A), which is paralleled by relatively large amounts of free SC in saliva (115) and an IgA level peaking in some studies temporarily at 4–6 weeks (111). These observations suggest that the newborn's salivary gland epithelium is under the influence of IFN-γ or other cytokines and microbial PRR/TLR stimulation to promote secretory immunity in this vulnerable period (24). Moreover, free SC in saliva may be of protective value by binding pIgA from breast milk to the oral epithelium of the nursed baby (116). Surprisingly, Cripps et al. (117) reported that saliva from one third of infants below 2.5 years contains only monomeric IgA. This is difficult to understand in view of the abundant perinatal epithelial expression of pIgR/SC (Fig. 13A) and J-chain expression as seen in virtually all salivary IgA^+^ PCs both before and after birth (46). In fact, Smith et al. (118) showed by molecular size analysis of IgA in 31 saliva from American infants that SIgA predominated, and a recent study reported this to be the case also in Estonian infants but remarkably not in Swedish infants (119). The authors had no explanation for this disparity except suggesting that the disparity might reflect geographical differences in epithelial PRR/TLR stimulation by microbial factors.

According to some studies, total IgM and IgM antibodies are often relatively prominent in saliva for the first postnatal months (47), but this is not a consistent finding (111). Partly because of the preferential external transfer of pIgA over pentameric IgM as discussed previously (Fig. 8A), infancy is rather characterized by a rapid increase of both IgA^+^ PCs (Fig. 13A) and salivary IgA levels which often peak before 2 months (47, 111). In the following few years, very little increase usually takes place (Fig. 13B), and most studies agree that adult salivary IgA levels are reached rather late in childhood (47, 114). On average, at the age of 6 months both the parotid IgA^+^ PC number and the salivary SIgA concentration have to increase approximately five times to reach the respective adult levels (Figs. 13A and 13B).

### Role of microbial exposure, breast feeding and nutrition

Geographical variations have a striking impact on the ontogeny of mucosal immunity. In contrast to the situation in Sweden, infants in a developing country exposed to poliovirus were often shown to have substantial levels of salivary IgA antibodies to poliovirus as early as 1 month after birth, generally approaching adult levels by the age of 6 months (120). Infants heavily exposed to *E. coli* from birth on, increased their salivary SIgA antibody levels significantly by 2 to 3 weeks of age, rapidly reaching adult levels (48). In less exposed infants, such levels were not attained until about 1 year of age, both for total SIgA and SIgA antibodies to *E. coli* O antigens (121). However, Swedish infants appeared to obtain increased salivary IgA antibody levels more readily in relation to hospitalization than when cared at home with regular breast feeding (121). A striking increase of salivary IgA in Australian children starting school was likewise ascribed to the environmental impact, particularly repeated respiratory tract infections (111).

The effect of breast feeding on salivary IgA levels in early infancy has been a matter of much dispute. The influence of growth factors, contamination of saliva with milk IgA, shielding of the secretory immune system in breast-fed infants by maternal SIgA antibody, and altered growth of the gut microbiota have been offered as alternative explanations for the discrepant observations. Most prospective studies have supported the notion that the early physiological increase of salivary IgA and IgM is more prominent in formula-fed than in solely breast-fed infants (47, 111). Observations in infants likewise suggest that breast feeding, in comparison with formula feeding, reduces the salivary IgA antibody levels to cow's milk proteins; this decrease was seen after a nursing period of only 3 weeks and appeared also in infants receiving mixed feeding (47, 111). Later on in infancy, however, the development of salivary IgA appears to be enhanced by breast feeding (114, 122).

A reduction of IgA in duodenal, nasal and salivary secretions has been observed in children with severe protein-calorie malnutrition (47). Such investigations have not distinguished between decreased production and reduced external transport of pIgA; adverse effects of vitamin A deficiency or infection on the pIgR/SC expression in secretory epithelia might cause the latter, but a study of mice rather suggested impairment of the local IgA response (123). Interestingly, a study of chronically undernourished children reported no reduction of total IgA in saliva, and the immune response (including salivary IgA antibodies) to oral vaccination with the B subunit of *V. cholerae* enterotoxin was similar to that of better-nourished children (124).

## Functions of antibodies in saliva

### Various defense mechanisms of SIgA

The remarkable stability of SIgA makes it well suited to function in protease-containing secretions such as whole saliva (125). Nevertheless, several oral bacteria produce enzymes that can selectively cleave SIgA1 in its extended (13-amino acid) hinge region, especially certain strains of *S. sanguinis* (previously *S. sanguis*) and *S. mitior* (previously *S. mitis*) but also *Porphyromonas/Prevotella* (previously *Bacteroides*) and *Capnocytophaga* species which are involved in periodontal disease (76). On average, at least 60% of salivary IgA consists of the IgA1 isotype (65), and parotid antibodies to *S. mutans* occur predominantly in this subclass, whereas reactivity to lipoteichoic acid from *S. pyogenes* and to lipopolysaccharides from *Porphyromonas gingivalis* (previously *Bacteroides gingivalis*), *B. fragilis* and *E. coli* is carried mainly by in the IgA2 isotype (126).

Although Fabα fragments released by the IgA1 proteases may retain antigen-binding capacity (127), this immune reaction may be adverse rather than protective. Such fragments may shield microorganisms from the defense function of SIgA antibodies and may even enhance epithelial colonization (128), whereas intact SIgA can specifically inhibit cellular attachment and penetration of influenza virus in contrast to monomeric IgA or IgG neutralizing antibodies (129).

**Fig. 14:**
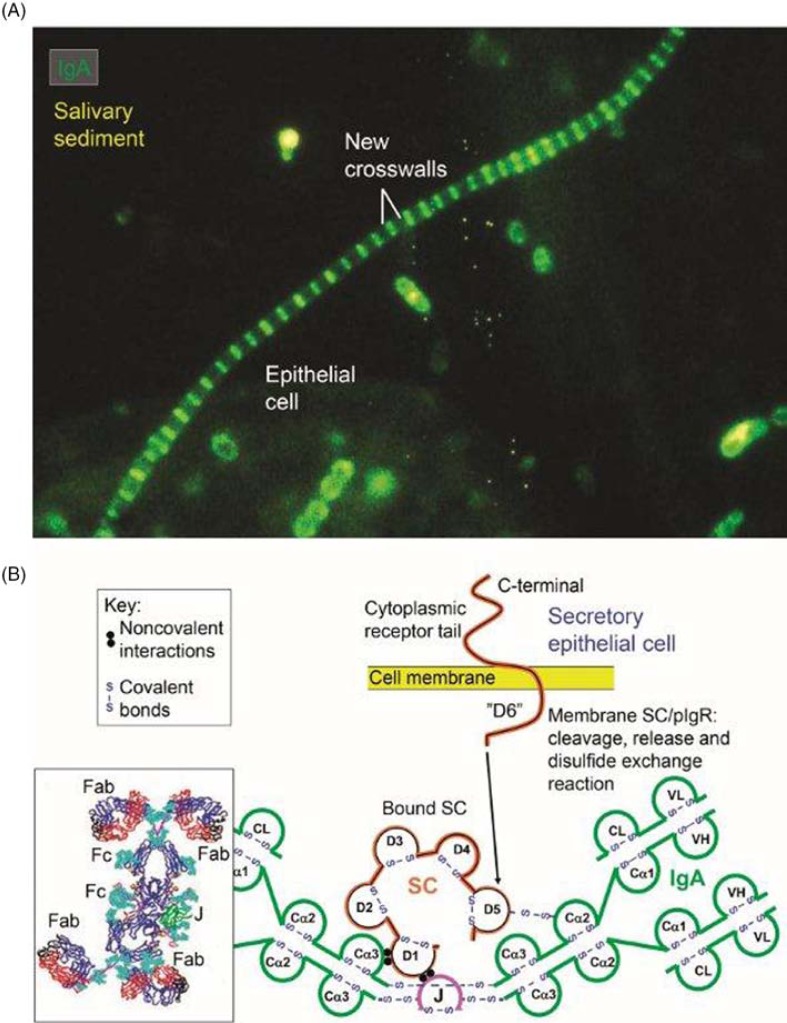
Synopses of functional properties of salivary IgA with the structural basis for the excellent antimicrobial binding activity of secretory IgA (SIgA). (A) Direct immunofluorescence staining of salivary sediment to demonstrate IgA adsorbed onto oral bacteria *in vivo*. Epithelial cell is faintly visualized because of autofluorescence. Numerous cocci (mainly diplococci) – partly adhering to epithelial cell – are coated with IgA, which is also bound to the older cell-wall segments of streptococci forming long chains, whereas new crosswalls formed *in vitro* after sampling are negative as indicated. Adapted from Brandtzaeg et al. (66) (original magnification: ×2000). (B) Domain interactions in the formation of SIgA based on data reviewed in Norderhaug et al. (29). Non-covalent domain interactions are shown between the J chain (J) and the extracellular domain 1 (D1) of bound SC, and covalent disulfide bonding is indicated between cystein 467 or 502 in D5 of SC and cystein 311 in the Cα2 domain of one of the two IgA subunits. Some studies have indicated that there may be two J chains in dimeric IgA (30). Insert to the lower left is from modeling data for dimeric IgA1 based on X-ray and neutron scattering in solutions published in Bonner et al. (137). Note the T-shape of the Fab fragments, allowing for antibody binding to large particles like bacteria. V, variable region; C, constant region; L, light chain; H, heavy chain; Fab, fragment antigen binding; Fc, fragment crystallizable.

The chief defense function of SIgA appears simply to be binding of soluble or particulate antigens in the action referred to as immune exclusion (130). *In vivo* coating of bacteria present in saliva with IgA can be directly demonstrated by immunostaining (Fig. 14A); and although this apparently does not inhibit bacterial growth (66), it is considered to provide containment of the microbiota and counteract invasiveness (Table 4). In mice, the IgA coating of gut bacteria was found to be unrelated to the total amount of SIgA exported to the intestinal lumen, suggesting that a specific reaction is involved (131). But bacterial IgA coating is no proof of antibody reactivity because many strains of group A or B streptococci possess Fcα receptors (132). Nevertheless, binding of SIgA to bacteria via Fc interactions may be of similar functional importance as Fab-mediated antibody coating, and the same regards microbial interactions with glycans of bound SC in SIgA (26). By affinity for mucin SIgA may also be involved in biofilm formation (133), but the putative role of this mechanism in dental plaque accumulation remains elusive. Interestingly, it was recently reported that mannose-containing oligosaccharides within human SIgA can alter virulence phenotype of *V. cholerae* such as biofilm formation (134).

**Table 4:** Antimicrobial effects of secretory IgA antibodies

SIgA is dimeric/polymeric and exhibits T-shaped Fab fragments, therefore exerting efficient microbial agglutination and virus neutralization SIgA performs non-inflammatory extracellular and intracellular immune exclusion by inhibiting epithelial adherence and invasion SIgA exhibits cross-reactive (‘innate-like’) activity as well as high-affinity somatic mutants and provides cross-protection in the herd SIgA (particularly SIgA2) is quite stable (bound SC stabilizes both isotypes of IgA) SIgA is endowed with mucophilic and lectin-binding properties (via bound SC in both isotypes and mannose in IgA2)

Modified from Brandtzaeg (130).

Many identified strategies may contribute to SIgA-mediated immune exclusion of antigens (130). In addition to more efficient antigen binding, complexing and neutralization (135), SIgA antibodies show better agglutinating properties than monomeric IgA, which may be aided by interaction with mucin in saliva (136). The combined effect of the dimeric structure and the T-shaped Fab fragments (137) – allowing SIgA antibodies to grasp big particulate antigens such as bacteria (Fig. 14B) – can largely explain the superior biological properties of SIgA antibodies (135). Their function appears to be further enhanced by a high level of cross-reactivity as observed for salivary IgA antibodies (138).

In line with the latter observation, at least one fourth of IgA^+^ PCs in human ileal lamina propria have been shown to produce polyreactive antibodies, which nevertheless were found to be somatically mutated with signs of antigen-driven selection (139). Other studies have shown that IgA autoantibodies produced in human duodenal mucosa are of high affinity but with little adaptation by somatic mutations, exhibiting mainly a germline repertoire (140). Notably in this context, recent mouse experiments suggested that there may be two fundamentally differentiation pathways for memory B cells – one dedicated to generation of high-affinity somatic antibody mutants, while the other preserves antibody germline specificities to arm the host for rapid responses to encountered variants of potentially dangerous antigens (141) – perhaps including the commensal microbiota. The latter immunological feature is characteristic for the IgA repertoire of human neonates (142), as also reflected in neonatal saliva (143). This situation is followed postnatally by a slow immune maturation with the somatic mutation frequency of IgA V_H_-gene transcripts reaching 25% of adult levels at the age of approximately 5 months (142). In fact, both the duodenal and the parotid frequency of IgA V_H_ mutations of adults is much higher than that in the adult human spleen, probably reflecting the constant antigenic pressure on the mucosal immune system (144). In the laboratory rat, however, a more restricted IgA repertoire (near germline) was revealed in salivary glands than in the distal small intestine (145).

Other recent mouse experiments demonstrated that the commensal coating with IgA in feces depends on appropriate clonal B-cell selection and affinity maturation in GALT germinal centers, and perhaps to some extent also in the lamina propria (146). In human feces some 40% of the anaerobic bacteria are normally coated with IgA (147) and this phenomenon can be observed in early childhood (148). Such IgA containment is probably important for the mutual host–microbe interaction, contributing to sustainable homeostasis by dampening proinflammatory signaling in the host and providing an immune pressure on commensal bacteria which results in antigenic drift without dysbiosis (146, 149). Altogether, however, it has to be admitted that many open questions remain about the mucosal IgA responses, both in mice and humans (150).

### Salivary immunoglobulins in relation to disease

Numerous studies have attempted to relate salivary IgA levels to a variety of oral as well as systemic diseases. Several reviews of such reports have been published but far from all available data can be considered conclusive (151–153). This is true also for studies of dental caries or periodontal disease in patients with primary immunodeficiencies, particularly selective IgA deficiency (154), although the results of early studies tended to suggest an inverse relationship between salivary IgA activity and caries susceptibility (151). Distinct results may be masked due to the fact that the immune defense operates in multiple layers so the possibility exists for various compensatory activities to come into play. Thus, it is well known that salivary SIgM is increased when SIgA is deficient (12), and the compensatory IgM reacts with commensal oral bacteria such as *S. mutans* (155). In mice it has been shown that adaptive and innate immunity cooperate flexibly to maintain host–microbiota mutualism (156); IgA deficiency will after some time be compensated by innate defense mechanisms which protect the gut epithelium from penetration of commensal bacteria (157).

#### Dental caries

How antibodies might protect against dental disease has for long been a matter of dispute, and both the role of salivary IgA and serum IgG in crevicular fluid have been considered (151, 152). As alluded to above, the balance of evidence indicates that there is an inverse relationship between caries susceptibility and the output of salivary IgA (151, 158), particularly in children and young adults (159). An inverse relationship between salivary IgA antibodies to *S. mutans* and its early oral colonization, or the colonized individual's caries experience, has also been reported; the mechanistic interest in this respect is focused on bacterial adhesions, glucosyl transferases, and glucan-binding proteins (160).

Parotid secretion of young adults regularly contains inhibiting IgA antibodies to glucosyltransferase, and this enzyme may play a major role in dental plaque formation. Oral vaccination with killed *S. mutans* of host origin induced salivary IgA antibodies that inhibited glucosyltransferase and reduced the numbers of viable *S. mutans* organisms in whole saliva and dental plaque (161). Also interestingly, an experimental study in young Americans suggested that a low parotid IgA antibody level to *S. mutans* serotype *c* was associated with enhanced colonization on molar tooth surfaces, whereas rapid clearance of serotype *d* was associated with relatively higher levels of corresponding parotid IgA antibodies (162).

Characterization of the salivary IgA response to *S. mutans* continues to be of interest (160) because of the high relevance of this bacterium in strategies aiming at a future active caries vaccine (163). A recent study of children at 3–4 years of age seemed to support this view because multivariate modeling showed that a lower baseline level of salivary IgA antibodies to glucan-binding protein was associated with higher caries risk (164). However, the authors pointed out that it is particularly difficult to study the secretory immune system in young children when it is undergoing significant maturation (163). In fact, a group of children, 8–12 years old, showed an increase of salivary IgA levels only in those with most caries lesions (165).

#### Periodontal disease

Several studies have shown a positive relationship between the concentration of salivary IgA (Table 2) and periodontal disease (13, 158, 159). Notably, elevated levels of parotid IgA antibodies to *Aggregatibacter* (previously *Actinobacillus) actinomycetemcomitans* were seen in subjects whose subgingival plaque harbored this microorganism (166). Other studies have likewise reported increased salivary IgA to *A. actinomycetemcomitans* and also to *P. gingivalis* patients with deep periodontal pockets (167). A possible link between the periodontal lesion and elevated IgA has been suggested to be production of IL-21 and IL-10 in the inflamed gingiva (168). However, accumulation of dental plaque may stimulate IgA production by increasing the amounts of swallowed bacteria that activate B cells in NALT and GALT.

Secretory immunity may hence be involved in host resistance to periodontal disease; but SIgA antibodies probably have little or no impact on the growth of an established dental plaque. Thus, an effect of accumulated plaque in an experimental gingivitis group of students was neither found on the total parotid IgA level nor on the IgA titer to a large number of oral bacteria, whether the subjects were smoking or not (169). Also, a more recent study of adults did not observe an effect of periodontitis on total salivary IgA levels, but found that smoking could adversely influence the level of IgA in saliva of the patients (170).

#### Airway infections and allergy

Most subjects with selective IgA deficiency in developed countries are clinically healthy, but some have increased tendency to allergy and infections, especially when compensatory production of SIgM is lacking (171). It has been reported that recurrent respiratory infections and isolation of pathogens are rarely seen in IgA-deficient children when salivary IgM is increased (172). This observation was subsequently supported by a study in adults with selective IgA deficiency; raised salivary levels of total IgM and of IgM antibodies to poliovirus and *E. coli* tended to be associated with relatively good resistance to infections of the respiratory tract (173). Proneness to such infections in selective IgA deficiency has been shown, moreover, to be associated with replacement of nasal IgA^+^ PCs mainly with cells producing IgG or IgD, whereas exclusive replacement with IgG^+^ and IgM^+^ PCs apparently confers satisfactory mucosal resistance (174).

In addition, several studies have been performed on subjects with no overt immunodeficiency to see if the level of salivary IgA shows a negative relationship to susceptibility to infection or allergy, mainly in the upper airways. Usually no clear-cut associations have appeared with infections (175), although infants born to atopic parents were reported to show significantly increased prevalence of reduced salivary IgA levels, presumably mainly being transient (176). Such temporary IgA deficiency was not convincingly related to later development of allergy, in agreement with a subsequent study where a significant relationship was found between transient salivary IgA deficiency and bronchial hyper-reactivity but not asthma (177). However, it is not known how a variable compensation with SIgM (which was not studied) could have influenced these results.

Other studies have reported that total salivary IgA tends to be reduced in infection-prone children with no overt immunodeficiency (178). This is in keeping with some other studies; but the possibility exists that the result could be explained by degradation of SIgA1 by microbial proteases. Salivary IgA may, moreover, be decreased in children with recurrent tonsillitis (109) or adenoid hyperplasia (110) and also, as alluded to above, in asthmatic children when the wheezing is precipitated mainly by recurrent respiratory tract infections (109). It has moreover been suggested that repeated antibiotic courses may lead to persistently low salivary IgA levels (179).

#### Human Immunodeficiency virus (HIV) infection and AIDS

Saliva of HIV-infected subjects may variably carry this virus (180), probably depending on the collection method and oral health, which both influence the number of HIV^+^ lymphocytes in the samples (181). Saliva from infected subjects contains IgA antibodies to HIV which to some extent may neutralize the virus, and there are HIV-inhibiting factors in saliva unrelated to antibodies (182).

Controversial results have been published concerning the effect of HIV infection on the salivary IgA level (182). Such inconsistency may be ascribed both to the disease stage and the sample source. Because HIV infection is often associated with elevation of serum IgA (mainly IgA1), it is important to avoid whole saliva for conclusive IgA studies, especially when the patients have candidiasis or other oral health problems. Atkinson et al. (183) first reported significantly elevated IgA in submandibular but not in parotid secretion from such patients, and Mandel et al. (184) confirmed the lack of parotid elevation. These observations might reflect that the IgA response in the submandibular/sublingual glands could be more closely related to GALT in terms of B-cell homing than that in the parotid, although the influence of a reduced flow rate on the salivary IgA level could not be excluded (183). Untreated AIDS patients do indeed have a highly upregulated intestinal IgA system (185), which might become disseminated to salivary glands.

Conversely, we showed that AIDS patients had reduced output of both parotid IgA1 and IgA2 (65). This observation was subsequently supported and extended to whole saliva (186). Also salivary IgA antibodies to viral p24 and gp160 are reduced in symptomatic patients (187). It is possible that in some studies, a leakage of monomeric IgA1 into whole saliva could have masked a decrease of locally produced salivary IgA1. Thus, when both the HIV infection status and the periodontal status are taken into account, the results of IgA measurements in whole saliva are difficult to interpret (188).

The level of IgG antibodies to p24 in whole saliva correlates with that in serum (187), again suggesting paracellular leakage from blood, either via gingival crevices (see earlier) or directly through the oral surface epithelium when touched by a sampling device – e.g. a swab. Tight junctions between epithelial cells are dynamic structures that rapidly open up under the influence of bacterial toxins and inflammatory mediators (189). It has been experimentally shown that IgG immune complexes increase leakage of bystander protein through rabbit sublingual epithelium, probably after activation of complement (190). Moreover, the slightest irritation of human nasal or intestinal mucosa leads to bulk flow of serum proteins to the epithelial surface (191). Also interestingly, salivary IgG has been reported to carry specific anti-gp160 activity 25-fold higher than that of serum IgG (192), suggesting some local production – perhaps in tonsils, inflamed gingivae, or submandibular and minor salivary glands, which contain a higher density of IgG^+^ PCs (Fig. 7) than the parotid glands (70, 73).

Adequately performed measurements of salivary IgG antibodies to HIV do show high specificity and sensitivity (193), and commercial kits are available for this purpose. Sampling of whole saliva has many advantages in scientific field studies of HIV infection and may be preferable to blood for antibody screening also in clinical settings – and particularly for home testing – although some issues concerning false positive and false negative results still need to be resolved (194).

## Conclusions

Oral microorganisms and aerodigestive antigens are continuously influenced by the two major antibody classes in saliva: SIgA and IgG. The former is synthesized as pIgA by PCs in salivary glands and is exported by an epithelial receptor-mediated mechanism. Conversely, most IgG occurring in saliva represent systemic immunity because it is derived from serum by passive diffusion – preferentially through gingival crevices – although a minor fraction may originate from glandular, gingival or tonsillar PCs. Along with the paracellular leakage of monomeric IgA and IgG antibodies, other serum-derived or locally produced factors and mediators will also appear in whole saliva such as IL-6 in patients with ulcerative colitis (195).

The secretory immune system is subject to complex regulation which influences distinctly the activity of the various cell types involved in SIgA formation. However, a number of immunological phenomena induced by mucosal antigen exposure are poorly defined in experimental animals and still more obscure in humans. There is evidence to suggest that GALT and NALT do not contribute equally to the induction of secretory immunity in various regions of the body. Because of such compartmentalization, it is unclear whether enteric immunization is the best way to stimulate salivary IgA responses. In fact, the various salivary glands may have different preferences.

In addition to possible compartmentalization of the oral secretory immune system, there are several other variables influencing the levels of total IgA and specific antibodies in salivary fluids. These include the impact of flow rate, protein loss during sample handling, difficulties with reproducibility and standardization of immunoassays, and uncontrolled admixture of serum-derived monomeric IgA and IgG to the samples. Nevertheless, salivary secretions still have interesting scientific and clinical potentials.
